# Signature-tagged mutagenesis screening revealed a novel smooth-to-rough transition determinant of *Salmonella enterica* serovar Enteritidis

**DOI:** 10.1186/s12866-017-0951-4

**Published:** 2017-03-03

**Authors:** Yang Jiao, Rongxian Guo, Peipei Tang, Xilong Kang, Junlei Yin, Kaiyue Wu, Shizhong Geng, Qiuchun Li, Jun Sun, Xiulong Xu, Xiaohui Zhou, Junji Gan, Xinan Jiao, Xiufan Liu, Zhiming Pan

**Affiliations:** 1 Jiangsu Key Laboratory of Zoonosis, Yangzhou University, 48 East Wenhui Road, Yangzhou, Jiangsu 225009 China; 2 Jiangsu Co-innovation Center for Prevention and Control of Important Animal Infectious Diseases and Zoonoses, Yangzhou, Jiangsu 225009 China; 3 Division of Gastroenterology and Hepatology Department of Medicine, University of Illinois at Chicago, 840 S Wood Street, Room 704 CSB, 60612 Chicago, IL USA; 4 Pathobiology and Veterinary Science, College of Agriculture, Health and Natural Resources, University of Connecticut, 61 North Eagleville Road, Unit-3089, Mansfield, CT USA

**Keywords:** *rfbG* gene, *S.* Enteritidis, Signature-tagged mutagenesis (STM), Smooth-to-rough transition, O_9_ MAb

## Abstract

**Background:**

*Salmonella enterica* serovar Enteritidis (*S*. Enteritidis) has emerged as one of the most important food-borne pathogens for humans. Lipopolysaccharide (LPS), as a component of the outer membrane, is responsible for the virulence and smooth-to-rough transition in *S*. Enteritidis. In this study, we screened *S.* Enteritidis signature-tagged transposon mutant library using monoclonal antibody against somatic O_9_ antigen (O_9_ MAb) and O_9_ factor rabbit antiserum to identify novel genes that are involved in smooth-to-rough transition.

**Results:**

A total of 480 mutants were screened and one mutant with transposon insertion in *rfbG* gene had smooth-to-rough transition phenotype. In order to verify the role of *rfbG* gene, an *rfbG* insertion or deletion mutant was constructed using λ-Red recombination system. Phenotypic and biological analysis revealed that *rfbG* insertion or deletion mutants were similar to the wild-type strain in growth rate and biochemical properties, but the swimming motility was reduced. SE Slide Agglutination test and ELISA test showed that *rfbG* mutants do not stimulate animals to produce agglutinating antibody. In addition, the half-lethal dose (LD_50_) of the *rfbG* deletion mutant strain was 10^6.6^ -fold higher than that of the parent strain in a mouse model when injected intraperitoneally.

**Conclusions:**

These data indicate that the *rfbG* gene is involved in smooth-to-rough transition, swimming motility and virulence of *S.* Enteritidis. Furthermore, somatic O-antigen antibody-based approach to screen signature-tagged transposon mutants is feasible to clarify LPS biosynthesis and to find suitable markers in DIVA-vaccine research.

## Background

*Salmonella enterica* serovar Enteritidis (*S.* Enteritidis, SE) has emerged as one of the most important food-borne pathogens for humans, with poultry meat and eggs being the most common sources of human *S.* Enteritidis food-borne infections [1]. Young chicks showed high mortality rate when infected with *S.* Enteritidis. However, in adult chickens, *S.* Enteritidis usually leads to symptomless carriage, and is able to colonize the tissues of the ovary and oviduct of egg-laying hens which result in egg contamination [2, 3]. Above all, *S.* Enteritidis constitutes a risk for public health.

In *Salmonella,* the lipopolysaccharide (LPS), as a significant component of the outer membrane, is responsible for virulence, smoothness and for mounting cross reactivity [4]. LPS is composed of three major structures—a core polysaccharide unit; the O-antigen, a polysaccharide consisting of repeating units of sugars that extend from the cell surface; and lipid A, a potent activator of the immune response, which anchors the LPS to the outer membrane [5]. Mutations in genes that are required for the synthesis of the LPS often result in a truncated LPS [4]. Mutant strains harboring incomplete LPS due to its truncation in the polysaccharide structure may have a smooth-to-rough transition [6]. Until now, some LPS deficient mutants (for instance, *rfaJ*, *rfaL* or *rfc.*) have been used to prevent infection caused by fowl typhoid, *Salmonella* Typhimurium and *Salmonella* Choleraesuis [4, 7, 8]. The purpose of this study was to identify novel factors that are involved in smooth-to-rough transition in *S.* Enteritidis.

Signature-tagged mutagenesis (STM) is a powerful tool to identify genes that are associated with a particular phenotype. The STM technique has been applied in several pathogens to identify conditionally essential genes during infection [9–11]. In previous studies in our laboratory, Geng et al. screened an STM bank of 1800 S. Gallinarum biovar Pullorum mutants and identified the genes essential for its survival in chickens. The attenuation of 10 mutants was confirmed by *in vivo* and in vitro competitive index (CI) studies. One highly attenuated *spiC* mutant was further characterized as a candidate vaccine [11].

Different serotypes of *Salmonella* have different O antigens. O antigens are used for serotyping of *Salmonella*. O9 antigen is one of the antigens produced on *Salmonella* Enteritidis. *Salmonella* Enteritidis with smooth phenotype have O9 antigen and therefore can be agglutinated by O9 antibody [12, 13]. However, *Salmonella* Enteritidis without O9 antigen would show rough phenotype. In this study, we used homemade monoclonal antibody against somatic O_9_ antigen (O_9_ MAb) [14] and commercial O_9_ factor rabbit antiserum to screen *S.* Enteritidis signature-tagged transposon mutants to identify novel factors involved in smooth-to-rough transition.

## Methods

### Bacteria, plasmids, primers and grow media

Bacterial strains, plasmids and primers used in this study are listed in the Table 1. Wild-type *Salmonella* Enteritidis strain C50041 was used in this study [15]. SE C50041*ΔspiC,* which was constructed by suicide plasmid in previous studies in our laboratory, was used as the recipient strain to make the mutant library in this study [16]. The plasmid pUT mini-Tn5Km2 (Cm) was constructed by inserting Cm^R^ gene into pUT mini-Tn5Km2. Bacteria were grown in LB broth (Difco). When needed, this medium was supplemented with 1.5% (w/v) Bacto-agar, ampicillin (Amp, 100 μg/ml), kanamycin (Km, 50 μg/ml) and chloromycetin (Cm, 40 μg/ml).

**Table 1:** Bacteria, plasmids and primers used in this study

Material	Description/purpose	Source or reference
Strains		
SE C50041	*Salmonella enterica* serovar Enteritidis; Wild-type; smooth	Hu et al., 2013 [15]
SE C50041*ΔspiC*	*ΔspiC* mutant of SE C50041; Recipient strain; smooth	Zeng et al., 2015 [16]
*E. coli.* χ7213-pir	Donor strain	Gift from R. Curtiss III
*E. coli.* DH5α	For cloning	Purchased from Takara company
SE C50041*ΔspiC* - *rfbG*::Tn5Km2(Cm)	*rfbG* transposon mutant of SE C50041*ΔspiC*	This study
SE C50041*ΔrfbG*	*ΔrfbG* mutant of SE C50041 by λ-Red recombination system	This study
*S.* Pullorum S06004	Negative control in the motility assay	Geng et al., 2009 [30]
Plasmids		
pUT mini-Tn5Km2(Cm)	For cloning	Constructed and stored in our laboratory
pKD3	Cm cassette template	Datsenko & Wanner, 2000 [17]
pKD46	λ-Red recombinase expression	Datsenko & Wanner, 2000 [17]
pCP20	FLP recombinase expression	Datsenko & Wanner, 2000 [17]
Primers		
Y linker	5’- CTGCTCGAATTCAAGCTTCT -3’	This study
P6U	5’- GAGCTCGAATTCGGCCTAG -3’	This study
*rfbG* forward	5’- AGGGCTGTGGGAAAAAGGTAAAGCTCCGTGGAAAACCTGGGAGTAAGTAGTGTGTAGGCTGGAGCTGCTTC -3’	This study
*rfbG* reverse	5’- CTCACGCAGGTTATTTGCTGTCATTACT TTGATTCCTTAAACTTATTTTCCATATGAATATCCTCCTTAG -3’	This study

### Construction of the transposon mutant library

According to the PCR-based STM working scheme, pUT mini-Tn5Km2(Cm) was used for transformation into *E. coli.* χ7213-pir, which required 2,6-diaminopimelic acid (DAP) for growth. The transformants were plated on selective LB agar plates containing Amp, Km, Cm and DAP. Thus the donor strain was generated. Conjugation was performed between the donor strain and the recipient strain SE C50041*ΔspiC* strain as described previously [11].

Briefly, 400 μl of the donor was mixed with 400 μl of the recipient. The mixture was immobilized on a 0.45 μm pore-size membrane filter placed on LB agar at 30 °C for 24 h. Transconjugants were recovered in 2 ml phosphate-buffered saline (PBS) and a 100 μl aliquot was plated on LB agar containing Km and Cm. All the potential conjugants were analysed for exclusive pUT mini-Tn5Km2(Cm) insertion by confirmation of Amp sensitivity. Each transconjugant was grown in a 96-well plate and stored in LB containing 20% glycerol at -80 °C for further use.

### Screening rough strains from mutant library

Frozen plates of the SE C50041*ΔspiC* transposon mutants were defrosted and subcultured by transferring 20 μl from each well to a new 96-well plate containing 180 μl of LB (containing Km and Cm). Plates were incubated overnight in a shaking incubator at 50 rpm at 37 °C. Subcultured strains were grown on LB agar containing Km and Cm at 37 °C for 16 h.

The slide agglutination tests were performed using O_9_ MAb developed previously in our laboratory and O_9_ factor rabbit antiserum (SSI®SALMONELLA ANTISERA, Denmark). AS handbook indicated, mutant culture from LB agar medium was mixed homogeneously with 1 drop of PBS and 1 drop of the O_9_ MAb or O_9_ factor rabbit antiserum on a glass slide. After the slide was tilted gently for approx. 1 min, the results were read.

Of the 480 colonies screened, a rough strain from primary screen was further verified by acriflavine agglutination test.

### Identification of transposon insertion site

Chromosomal DNA was isolated from transposon mutant and completely digested with *Nla*III that cut on either end of the transposon. Meanwhile, an adapter of a double-stranded cassette was generated as described previously [11]. Approximately 80 ng purified DNA from the digested DNA was ligated to 1 μg of the adapter using a DNA ligation kit (Takara, Dalian, China) in 10 μl at 16 °C for 12 h. The reaction mixture was diluted with double distilled water to 100 μl as templates in the PCR amplification. Sequencing of DNA flanking the transposon was done by Y linker and P6U primer (Table 1). DNA sequence flanking the transposon insertion site was identified by BLAST-N alignment with the recently sequenced SE P125109 in the NCBI GenBank database (GenBank accession NO. AM933172.1).

### Construction of the *rfbG* gene deletion mutant

The knock-out mutant, SE C50041*ΔrfbG* was constructed by λ-Red recombination system [17]. Briefly, the *rfbG* gene was first substituted by a PCR adjusted antibiotic resistance cassette (Cm) using a λ-Red helper plasmid pKD46, which encode a series of phage recombinase. Recombinant clones were selected by plating on LB agar containing Cm. To resolve the antibiotic resistance cassettes (Cm), the temperature sensitive plasmid pCP20 was introduced. Finally, the *rfbG* gene was completely deleted from the start codon through the stop codon, as confirmed by sequencing. The slide agglutination tests were performed as above to determine whether SE C50041*ΔrfbG* was rough strain.

### Auto-aggregation assay LPS and SDS-PAGE silver staining of the mutants

The auto-aggregation assay was performed based on the method previously described by Zhou et al. [18]. Briefly, Salmonella cultures were statically grown in 5 ml LB medium at 37 °C for 16 h in test tubes. The upper 0.2 ml was carefully removed to measure its optical density (OD_600)_ (recorded as OD_600_ prevortex). The remaining culture in the test tube was then mixed by vortexing to re-suspend the aggregated cells, and 0.2 ml of the suspension was removed and its OD_600_ was measured (recorded as OD_600_ postvortex). The “percent aggregation” was calculated using the formula: 100% *(OD_600_ postvortex - OD_600_ prevortex)/OD_600_ postvortex.

Validation of the LPS phenotype occurred by SDS-PAGE and silver staining [19]. For this purpose LPS was isolated from SE C50041 and SE C50041*ΔrfbG* using a commercially available LPS extraction kit (Intron biotechnology, Gyeonggi-do, Korea). The obtained LPS was separated by standard SDS-PAGE and was stained using a pierce® silver stain kit (Thermo, Rockford, USA).

### Analysis of in vitro growth and biochemical characteristics of the mutants

For in vitro growth analysis of SE C50041, SE C50041*ΔspiC*, SE C50041*ΔspiC* - *rfbG*::Tn5Km2(Cm) and SE C50041*ΔrfbG*. A single colony of each strain was subcultured in 5 ml LB broth and cultured at 37 °C with shaking at 180 rpm for at least 12 h. Subsequently the absorbance value of each strain was determined by spectrophotometry and cultures were diluted in 20 ml LB broth, then the absorbance value was determined by spectrophotometry to achieve an approx. initial concentration (OD_600_ = 0.05) as a starting time point (0 h). The cultures were incubated at 37 °C with shaking at 100 rpm and the OD_600_ was determined at time points of 0.5, 1, 1.5, 2, 2.5, 3, 4, 5, 6, 7, 8, 9, 10, 11, 12 and 13 h. Each strain was tested in triplicate in two independent experiments. Biochemical traits of strains were tested by VITEK® 2 microbial identification system (bioMérieux, Marcy l'Etoile, France), including glucose, maltose, sucrose, mannose, mannitol, lactose, dulcitol, adonitol, sorbitol, malonate, lysine decarboxylase, ornithine decarboxylase, urea, H_2_S and so on.

### Motility Assay

LB plates containing 0.3% (w/v) agar was used to characterize the motility phenotype of SE C50041, SE C50041*ΔspiC*, SE C50041*ΔspiC* - *rfbG*::Tn5Km2(Cm) and SE C50041*ΔrfbG*. Overnight cultures of each strain were adjusted to the same optical density. Equal volume of suspensions were incubated on spots onto 0.3% LB agar. The plates were incubated at 37 °C for 5 h, and motility was assessed by examining the migration of the bacteria from the center of the inoculation point to the periphery of the plate [20]. The data were representative of three independent experiments, which gave similar results.

### Preparation and identification of sera

The SPF chickens were obtained from Poultry Institute of Shandong Academy of Agricultural Science and the chickens were detected for free from any clinical signs of enteric disease and negative for *Salmonella*. All chickens were given formulated commercial feed and water throughout the experimental period. Experiments were undertaken in accordance with the permission of the Animal Care and Ethics Committee of Yangzhou University.

Three-week-old chickens were inoculated intramuscularly with 100 μl of bacteria suspended in PBS solution and then a boost on day 14 after the first immunization, each bacteria inoculum was 1 × 10^8^ CFU (intramuscularly, *n* = 5). Chickens were immunized respectively with SE C50041*ΔspiC*, SE C50041*ΔspiC* - *rfbG*::Tn5Km2(Cm) or SE C50041*ΔrfbG*. A group of chickens was also infected with wild-type strain SE C50041. Five control chickens received 100 μl of PBS via the same route. Sera from each animals (20 μl) were mixed with some SE C50041 and observed for agglutination reaction [4].

The flocktype® *Salmonella* Ab ELISA kit (QIAGEN, Leipzig, Germany) was also used to determine the presence of serum antibody to the O-antigens 1, 4, 5, 9, and 12 as handbook indicated. An S/P ratio of ≥0.3 was considered positive while <0.2 was considered negative, samples with the S/P ratio ≥0.2 and <0.3 are doubtful.

### Virulence assessment

BALB/c mice were obtained from the experimental animal centre of Yangzhou University. The mice were housed in an animal facility under a standard animal study protocol. Experiments were undertaken in accordance with the permission of the Animal Care and Ethics Committee of Yangzhou University.

To investigate the virulence of SE C50041*ΔrfbG* in BALB/c mice (6 - 8 weeks of age), Two groups of mice (each containing 25 mice) were infected with SE C50041*ΔrfbG* and SE C50041. Mice in each group were further subdivided into five subgroups, each containing five mice. Each mouse in the C50041*ΔrfbG* group was injected intraperitoneally with 10-fold dilutions of the strain from 1 × 10^8^ - 1 × 10^4^ CFU in 100 μl PBS. Each mouse in the C50041 groups was injected intraperitoneally with 10-fold dilutions of the strain from 1 × 10^4^ - 1 × 10^0^ CFU in 100 μl PBS. Five control mice received 100 μl of PBS via the same route. Deaths were recorded up to day 14 and the LD_50_ of each strain was calculated using the Karber and Behrens method [21].

### Statistical analysis

All statistical analyses were performed using GraphPad Prism. *P* values < 0.05 were considered significant when using one-way analysis of variance (ANOVA).

## Results

### Identification of mutant *S.* Enteritidis showing smooth-to-rough phenotype

**Fig. 1:**
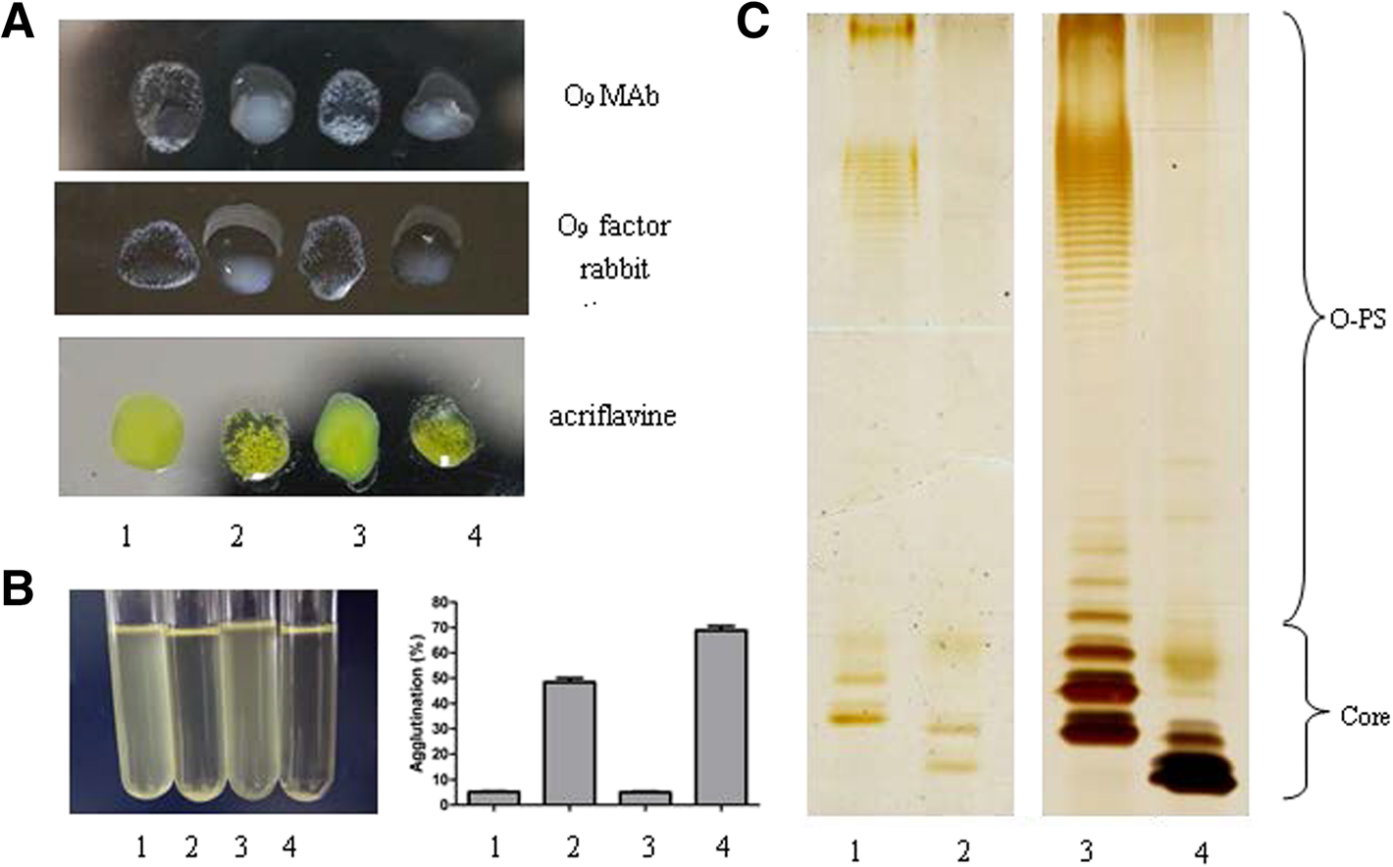
Rough strain characteristics of *ΔrfbG* mutants. **a** SDS-PAGE with silver staining of LPS of *ΔrfbG* mutants (SE C50041*ΔrfbG* and SE C50041*ΔspiC* - *rfbG*::Tn5Km2(Cm)) compared to SE C50041 or SE C50041*ΔspiC*. **b** Agglutination was examined with O_9_ MAb, O_9_ factor rabbit antiserum and acriflavine. Pictures were taken within 5 min. **c** Visual aggregation and “percent aggregation” of *ΔrfbG* mutants (SE C50041*ΔrfbG* and SE C50041*ΔspiC* - *rfbG*::Tn5Km2(Cm)), SE C50041 and SE C50041*ΔspiC* cultures grown statically for 16 h at 37 °C. 1, SE C50041; 2, SE C50041*ΔrfbG*; 3, SE C50041*ΔspiC*; 4, SE C50041*ΔspiC* - *rfbG*::Tn5Km2(Cm)

Out of 480 mutants screened, 1 potential mutant was not agglutinated with O_9_ MAb or O_9_ factor rabbit antiserum. To confirm that the transposon mutant had a rough phenotype, an acriflavine agglutination test was performed and the result showed that this strain was strongly agglutinated with acriflavine (Fig. 1a).

The sequence of the DNA flanking the transposon insertion site from this rough mutant was identified to be *rfbG* (region from 2176901 to 2177980 in SE P125109), which encodes a putative CDP-glucose 4,6-dehydratase.

### Deleted mutant *ΔrfbG* of *S.* Enteritidis becoming rough pattern

The deletion mutant *ΔrfbG* of *S.* Enteritidis was constructed by λ-Red recombination system. The slide agglutination tests showed that SE C50041*ΔrfbG* was not agglutinated with O_9_ MAb or O_9_ factor rabbit antiserum, but was agglutinated with acriflavine (Fig. 1a). SE C50041*ΔrfbG* also demonstrated smooth-rough transition.

### Auto-aggregation of *ΔrfbG* mutants and SDS-PAGE silver staining of LPS

The auto-aggregation was tested in Luria-Bertani (LB) broth (Fig. 1b) and the “percent aggregation” was calculated using OD_450_ measurements from these cultures. Both the SE C50041 and SE C50041*ΔspiC* showed 5% aggregation, then the SE C50041*ΔrfbG* and SE C50041*ΔspiC* - *rfbG*::Tn5Km2(Cm) demonstrated 48% and 69% aggregation, respectively (Fig. 1b).

LPS patterns obtained by standard Sodium dodecyl sulfate-polyacrylamide gel electrophoresis (SDS-PAGE) of SE C50041, SE C50041*ΔspiC*, SE C50041*ΔspiC* - *rfbG*::Tn5Km2(Cm) and SE C50041*ΔrfbG* are presented in Fig. 1a. It showed a visible loss of O-antigens and Core-LPS antigens for *ΔrfbG* mutants (SE C50041*ΔrfbG* and SE C50041*ΔspiC* - *rfbG*::Tn5Km2(Cm)) compared to SE C50041 or SE C50041*ΔspiC*. There was no obvious difference between *ΔrfbG* mutant and SE C50041*ΔspiC* - *rfbG*::Tn5Km2(Cm) (Fig. 1c).

### Growth and biochemical characteristics of *ΔrfbG* mutants

**Fig. 2:**
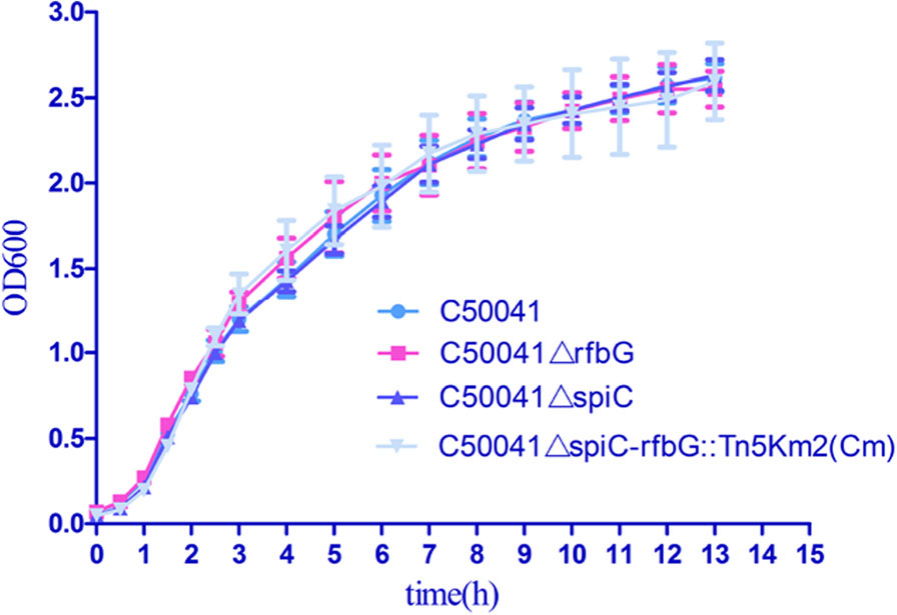
Growth curves of *ΔrfbG* mutants. The SE C50041, SE C50041*ΔrfbG*, SE C50041*ΔspiC* and SE C50041*ΔspiC* - *rfbG*::Tn5Km2(Cm) showed an identical growth response in LB broth

Growth curve analysis revealed no significant differences between the wild-type and each mutant when cultured in LB broth at 37 °C (Fig. 2). Results of biochemical tests including glucose, maltose, sucrose, mannose, mannitol, lactose, dulcitol, adonitol, sorbitol, malonate, lysine decarboxylase, ornithine decarboxylase, urea, H_2_S and so on were the same between wild-type and each mutant, suggesting mutations in these genes do not alter the biochemical characteristics of *S.* Enteritidis.

### Motility of *ΔrfbG* mutants

**Fig. 3:**
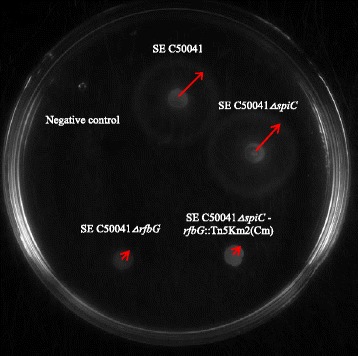
Motility assay of *ΔrfbG* mutants. Motility assay of the SE C50041, SE C50041*ΔspiC*, SE C50041*ΔspiC* - *rfbG*::Tn5Km2(Cm) and SE C50041*ΔrfbG. S.* Pullorum S06004, was used as a negative control on swimming agar. After 5 h of incubation at 37 °C on swimming plates, the semi-diameter of each bacterial growth area was measured. These results shown are representative of three independent experiments. The migrations of strains are highlight using *red arrows*

Motility plates (Fig. 3) displayed a marked reduction in migration from the inoculation site to the periphery of the plate for SE C50041*ΔrfbG* (2.5 mm) and SE C50041*ΔspiC* - *rfbG*::Tn5Km2(Cm) (3.0 mm) when compared to SE C50041 (15.5 mm) and SE C50041*ΔspiC* (14.5 mm). The data were representative of three independent experiments, which gave similar results.

### Sera tests

Sera samples were collected at 14 days after the second immunization with SE C50041*ΔspiC*, SE C50041*ΔspiC* - *rfbG*::Tn5Km2(Cm), SE C50041*ΔrfbG*, SE C50041 or PBS control. Sera collected from SE C50041*ΔspiC* - *rfbG*::Tn5Km2(Cm), SE C50041*ΔrfbG* and control groups were not agglutinated with SE C50041. In contrast, sera collected from the other two groups showed obvious reaction (Table 2).

**Table 2:** Positive rate of sera tests (agglutination and ELISA)

Strains	Agglutination	ELISA
SE C50041	5/5	5/5
SE C50041*ΔspiC*	5/5	5/5
SE C50041*ΔrfbG*	0/5	0/5
SE C50041*ΔspiC* - *rfbG*::Tn5Km2(Cm)	0/5	0/5

Sera samples of chicks immunized with *ΔrfbG* mutants (SE C50041*ΔspiC* - *rfbG*::Tn5Km2(Cm) (*n* = 5) or SE C50041*ΔrfbG* (*n* = 5)) and control animals (*n* = 5) were considered *Salmonella* negative when using a commercially available flocktype® *Salmonella* Ab ELISA kit. Chicks immunized with SE C50041*ΔspiC* (*n* = 5) or SE C50041 (*n* = 5) were considered seropositive for *Salmonella* in the ELISA test (Table 2). In conclusion, *ΔrfbG* mutants without O-antigen do not stimulate animals to produce relevant antibody.

### Virulence of *ΔrfbG* mutant

To investigate the role of *rfbG* on the virulence, mice were injected intraperitoneally with SE C50041*ΔrfbG* and SE C50041, and half-lethal dose (LD_50_) values were calculated according to the method of Karber and Behrens. The LD_50_ of *ΔrfbG* was 10^6.69^, which was 10^6.6^ -fold higher than that of SE C50041(10^0.13^), implying that the virulence of the SE C50041*ΔrfbG* was significantly decreased (*P* < 0.05).

## Discussion

For gram-negative bacteria, including *Salmonella*, LPS are essential components of immunodominant antigens. For *Salmonella*, some LPS deficient mutants represent a promising research area. These mutants can not only result in attenuation, but also show structural (smooth-rough) transition that can be used as a marker for distinguishing isolates [19]. Therefore, the present study was performed to identify novel factors of *S.* Enteritidis that are important for smooth-to-rough transition.

STM is a powerful genetic tool that allows identification of genes that are important for different facets of pathogenesis and is well suited for screening rough strain in vitro. In this study, we first attempted to use O_9_ MAb and O_9_ factor rabbit antiserum to screen for signature-tagged transposon mutants of *S.* Enteritidis with slide agglutination tests. We found that *rfbG* gene was involved in smooth-to-rough transition in *S.* Enteritidis. Meanwhile, we also identified some other genes that involved in smooth-to-rough transition, e.g., *rfc* (data not shown), which has been used as a marker for distinguishing in *Salmonella enterica* vaccine research [22, 23]. In line with this, this new approach of using O_9_ MAb and O_9_ factor rabbit antiserum to screen *S.* Enteritidis signature-tagged transposon mutants to identify novel loci involved in smooth-to-rough transition is useful and reliable.

The *rfb* gene cluster of *S.* Typhimurium contains genes that are responsible for all or part of the biosynthetic pathways of dTDP-L-rhamnose, ODP-abequose and GDP-mannose, and are essential for O-antigen biosynthesis. Among them, the *rfbG* gene, which encodes a CDP-glucose 4,6-dehydratase, is a component of abequose biosynthetic pathway. CDP-glucose 4,6-dehydratase and glucose-1-phosphate cytidylyltransferase (*rfbF*) are two enzymes required to promote the formation of CDP-4-keto-3,6-dideoxyglucose from CDP-4-keto-6-deoxyglucose (*rfbH* and *rfbI*), and abequose synthase (*rfbJ*) [24]. On the basis of amino-acid sequence homology, *S.* Typhi CDP-glucose 4,6-dehydratase is known to be a member of the short-chain dehydrogenase/reductase(SDR) superfamily, with the N-terminal domain contains a Rossmann fold and provides the platform for NAD(H) binding. The C-terminal domain is composed mostly of α-helix and houses the bingding pocket for the CDP portion of the CDP-xylose ligand. The xylose moiety extends into the active-site cleft that is located between the two domains [25]. It has also been demonstrated that the *rfbG*-negative *Azotobacter vinelandii* grown in liquid medium exhibited agglutination, suggesting that *rfbG* gene is involved in surface structural transition [26].

It has been described that some LPS deficient mutants (*rfaJ*, *rfaL*, *rfc*, *et al.*) were used in *Salmonella* DIVA-vaccine (Differentiation of Infected and Vaccinated Animals) research [19, 27, 28]. In the present study, the virulence change of the *rfbG* mutant strain were measured in mouse model. The result demonstrated that *rfbG* mutant strain is safe to mammal. Furthermore, SE Slide Agglutination test and ELISA test showed that *rfbG* mutants do not stimulate animals to produce agglutinating antibody, which may help to distinguish animals vaccinated with this mutant from those infected by field strains. Overall, *rfbG* mutant showed a potential “DIVA” capacity. Nevertheless, as a candidate vaccine, *rfbG* mutant needs further study.

In addition, results obtained from motility assays indicate that the deletion of *rfbG* gene resulted in variation in swimming motility, suggesting that flagellar assembly and function may be influenced by the altered LPS structures. Deditius et al. described that a *rfaG* mutant diminished flagellar assembly and significantly reduced transcription of flagellar class II and class III promoters, but not of the class I promoter. Moreover, FlhC protein levels were reduced in the *rfaG* mutant strain. They concluded that a defect in LPS biosynthesis regulates motility by affecting FlhDC stability or translation of its mRNA on a posttranscriptional level via an unknown mechanism [29]. Moreover, the results obtained from SDS-PAGE silver staining of LPS show that *ΔrfbG* mutants not only lead to loss of O-antigens but also lead to loss of Core-LPS antigens. It suggested that deficiency in *rfbG* leads to deep loss of LPS synthesis. The mechanism of *rfbG* affect LPS biosynthesis need further studies.

## Conclusions

We used O_9_ MAb and O_9_ factor rabbit antiserum to screen *S.* Enteritidis signature-tagged transposon mutants to identify novel factors involved in smooth-to-rough transition. The present study demonstrated that *rfbG* gene inserted/deletion mutant of *S.* Enteritidis showed almost the same biological characteristics, attenuation, distinguishable reaction (agglutination and ELISA) and other rough strain characteristics. Thus, this approach may be used more broadly in exploring LPS biosynthesis, and as a high-throughput tool for screening rough strains which were used as markers in developing DIVA-vaccine.

## Abbreviations

Amp: Ampicillin
ANOVA: One-way analysis of variance
CI: Competitive index
Cm: Chloromycetin
DAP: 2,6-diaminopimelic acid
Km: Kanamycin
LB: Luria-Bertani
LD_50_: Half-lethal dose
LPS: Lipopolysaccharide
O_9_ MAb: Monoclonal antibody against somatic O_9_ antigen
PBS: Phosphate-buffered saline
*S.* Enteritidis SE: *Salmonella enterica* serovar Enteritidis
SDS-PAGE: Sodium dodecyl sulfate-polyacrylamide gel electrophoresis
STM: Signature-tagged mutagenesis
